# Mannose-Binding Lectin Inhibits the Motility of Pathogenic *Salmonella* by Affecting the Driving Forces of Motility and the Chemotactic Response

**DOI:** 10.1371/journal.pone.0154165

**Published:** 2016-04-22

**Authors:** Jun Xu, Shuichi Nakamura, Md. Shafiqul Islam, Yijie Guo, Kohei Ihara, Rintaro Tomioka, Mizuki Masuda, Hiroshi Yoneyama, Emiko Isogai

**Affiliations:** 1 Department of Animal Microbiology, Graduate School of Agricultural Science, Tohoku University, Sendai, Miyagi, Japan; 2 Department of Applied Physics, Graduate School of Engineering, Tohoku University, Sendai, Miyagi, Japan; 3 Department of Immunobiology and Pathogenic Biology, Medical School of Xi’an Jiaotong University, Xi’an, China; University of Leicester, UNITED KINGDOM

## Abstract

Mannose-binding lectin (MBL) is a key pattern recognition molecule in the lectin pathway of the complement system, an important component of innate immunity. MBL functions as an opsonin which enhances the sequential immune process such as phagocytosis. We here report an inhibitory effect of MBL on the motility of pathogenic bacteria, which occurs by affecting the energy source required for motility and the signaling pathway of chemotaxis. When *Salmonella* cells were treated with a physiological concentration of MBL, their motile fraction and free-swimming speed decreased. Rotation assays of a single flagellum showed that the flagellar rotation rate was significantly reduced by the addition of MBL. Measurements of the intracellular pH and membrane potential revealed that MBL affected a driving force for the *Salmonella* flagellum, the electrochemical potential difference of protons. We also found that MBL treatment increased the reversal frequency of *Salmonella* flagellar rotation, which interfered with the relative positive chemotaxis toward an attractive substrate. We thus propose that the motility inhibition effect of MBL may be secondarily involved in the attack against pathogens, potentially facilitating the primary role of MBL in the complement system.

## Introduction

The innate immune protein mannose-binding lectin (MBL) is a collagenous lectin found in the serum of warm-blooded animals, and plays a crucial role in the first line of defense against infections [1]. MBL belongs to the C-type lectin family, which all possess a carbohydrate-recognition domain. MBL interacts with mannose-rich residues present on the pathogen surface in a Ca^2+^-dependent manner. The association of MBL with pathogens then promotes the opsonization of pathogens and subsequent activation of phagocytic cells via initiating the lectin pathway of the complement system, which triggers the formation of C3b and a membrane attack complex against the pathogens. Abnormally low level of MBL in human leads to a defect of opsonization, resulting in a high risk of infections by viruses [2], parasites [3], and bacteria [4, 5].

The well-known bacterial pathogen *Salmonella* causes food-borne gastroenteritis to humans and other animals. *S*. *enterica* is a major zoonotic pathogen and has been classified into several subspecies. These subspecies are further subdivided into serovars, which are differentiated by the structures of their flagellum, carbohydrates, and lipopolysaccharides (LPS) [6, 7]. The strains of *Salmonella* are known to highly express mannose-rich LPS, and the mechanism of binding between MBL and their LPS has been investigated [5, 8]. For example, Kuhlman *et al*. [5] demonstrated the binding of MBL to a *Salmonella* strain producing a mannose-rich LPS using a fluorochrome conjugated anti-MBL serum. Studies on the effect of MBL binding to *Salmonella enterica* serovar Typhimurium have revealed the bactericidal properties of MBL [9, 10].

The virulence of *Salmonella* is enhanced by their motility [11, 12]. *Salmonella* strains are peritrichous, and their propulsive forces for swimming are generated by rotation of multiple external flagella. Each flagellar filament is linked with a flagellar motor embedded in the inner membrane, which is driven by an electrochemical potential difference of protons called the proton motive force (PMF) [13, 14]. The flagellar motor can spin in both a counter-clockwise (CCW) and clockwise (CW) direction. Although all of the flagella tend to rotate CCW, the flagella form a tail-like bundle, which smoothly propels the cell forward. The bundle is deformed when some of the flagella switch their rotational direction to CW, so that the cell transiently tumbles [12, 13, 15–17]. Because the cell can randomly change the direction of a cell-body long axis while the cell tumbles, repeated swimming and tumbling allows the cell to display a biased-random motion, enabling it to stochastically migrate toward favorable environments and to move away from unfavorable environments [18].

The correlation between motility and pathogenicity has been reported in other bacterial species besides *Salmonella* [19–21]. For example, the motility of *Leptospira*, a pathogenic spirochete causing worldwide zoonosis leptospirosis outbreaks, is also known as a critical factor for establishing infection [22]. Mannose is a sugar component of the LPS in some *Leptospira* strains [23], and our recent study showed that MBL impairs the motility of *Leptospira* [24]. Thus, MBL is considered to contribute to preventing infection of microorganisms through not only opsonization but also by inhibiting the motility of pathogens. However, precisely how the association of MBL with cells affects their motility remains unknown.

To understand the mechanism of motility inhibition by MBL, we investigated the effect of MBL on energetic parameters required for motility and chemotaxis in *Salmonella* strains. We show that MBL inhibits the motility of *Salmonella* by affecting the driving forces for the flagella. We also show that the association of MBL with *Salmonella* cells interferes with their chemotaxis toward an attractant due to an aberrant reversal frequency of the flagella.

## Materials and Methods

### Bacterial strain and media

*Salmonella enterica* serovar Typhimurium strains st1wt [25] and SJW3076 [Δ(*cheA*–*cheZ*)] [26] were used in this study. The strain st1wt has intact flagella and a chemotaxis system. The strain SJW3076 has intact flagella but lacks a chemotaxis system. The strains were cultured in L-broth, containing 10 g of tryptone (Nacalai Tesque, Kyoto, Japan), 5 g of yeast extract (Nacalai Tesque), and 5 g of NaCl per liter. The motility buffer contained 10 mM potassium phosphate, 0.1 mM ethylenediaminetetraacetic acid, and 10 mM sodium lactate [27]. Human MBL (R&D Systems, Minneapolis, MN) was diluted with phosphate buffered saline containing 5 mM CaCl_2_, because the binding of MBL is known to occur in a bivalent calcium ion-dependent manner [28, 29]. The physiological concentration of MBL is about 2.5 μg/ml in humans [30]. For comparison, bovine serum albumin (Sigma-Aldrich, MO), anti-*Salmonella* O antigen serum (DENKA SEIKEN, Tokyo, Japan), and the plant lectin protein peanut agglutinin (PNA) (Sigma-Aldrich, MO) were used.

### Analysis of the motile fraction and swimming speed

Cells were grown in L-broth at 37°C until reaching the early stationary phase. The cell culture was diluted 1:20 in the motility buffer and then the cells were treated with various concentrations of proteins serially diluted with phosphate buffered saline. Ten microliters of the solution was infused into a flow chamber composed of a coverslip (upper side) and a glass slide (bottom side) for observations of motility under a dark-field microscope (BH2, Splan 40×, NA 0.70; Olympus, Tokyo, Japan), and motility was recorded on a DVD by a charge-coupled device (CCD) camera (SSC-M350; Sony, Tokyo, Japan) at a frame rate of 30 frames per second. The movies were captured on a computer as AVI files. Swimming trajectories and the speeds of individual cells were analyzed using ImageJ software (National Institutes of Health, Bethesda, MD, USA) and VBA-based macros programmed in Microsoft Excel (Microsoft, Bellevue, WA, USA) [31].

### Rotation assay for a single flagellum

Rotation of the *Salmonella* flagellar motor was analyzed by a tethered-cell assay [32, 33]. Five hundred microliters of the cell suspension was centrifuged at 8000 ×*g* for 2 min and the supernatant was removed. The cells were suspended in the motility buffer and their flagellar filaments were sheared by passing the cell suspension through a needle (27 G) using a 1-mL syringe in order to reduce the number of flagella in each cell and enable rotation assays for a single flagellum. An immune serum containing an antibody against *Salmonella* flagellum (H antigen) (DENKA SEIKEN, Tokyo, Japan) was infused into a flow chamber and incubated at room temperature for 5 min to adhere to the antibodies on the glass. Unbound antibodies were washed away with the motility buffer, and the cell suspension was infused and incubated at room temperature on the stage of the dark-field microscope until rotating cells were observed. The cell rotation was observed through an objective (Splan 40×, NA 0.70; Olympus) and recorded with a high-speed CCD camera (ICL-B0620M-KC, IMPERX, FL, USA) at 10-ms intervals. Analysis of the tethered-cell rotation was performed using ImageJ and Microsoft Excel macros.

### Measurement of intracellular pH

The change in the intracellular pH of *Salmonella* was examined by using enhanced green fluorescent protein (EGFP) [34]. The absorption spectra of EGFP vary depending on pH [34]. *Salmonella* strain st1wt cells carrying a plasmid encoding the *EGFP* gene (vector pEGFP, GenBank accession no. U76561; Clontech, Mountain View, CA, USA) were grown overnight in L-broth containing 100 μg/ml ampicillin at 37°C. One milliliter of the culture was washed twice with the motility buffer by centrifugation (8000 ×*g* for 2 min). The cells were suspended in 500 μl of the motility buffer. After the addition of 2.5 μg/ml MBL or 20 mM potassium benzoate (pH 5) (Wako, Osaka, Japan), the fluorescent absorption spectra were measured with a fluorescence spectrophotometer (F-7000; Hitachi, Tokyo, Japan) at 509 nm with excitation at 490 nm at room temperature. Triplicate experiments were performed in each condition.

### Measurement of membrane potential

The membrane potential was measured by the anionic membrane-potential sensitive fluorescent probe DiBAC_4_(3) (Dojindo Laboratories, Japan) [35]. Cells were grown in L-broth at 37°C until the early stationary phase. The culture was diluted to 10^6^ cells/ml and incubated with 10 μg/ml DiBAC_4_(3) for 10 min, distributing the dye over the membrane surface. Subsequently, 2.5 μg/mL MBL, or 20 μM valinomycin and 150 mM KCl was added to the cell suspension containing DiBAC_4_(3) at the external pH of 7.0 [36–38], and the fluorescence intensity at 517 nm obtained by excitation at 495 nm was determined using a fluorescence plate reader (PowerScan HT, DS Pharma Biomedical, Osaka, Japan).

### Microscopic agar drop assay

A flow chamber was constructed as previously described [39]. A drop of 2% agar (Wako) containing 10 mM serine (Wako), which serves as an attractant for *Salmonella*, was placed around the center of the flow chamber. An overnight culture of *Salmonella* was diluted to 1:10 in the motility buffer with or without MBL and infused into the flow chamber. Images of motile cells near the agar drop were recorded by a dark-field microscope (BX50, Splan 40×, NA 0.70; Olympus) and CCD camera (SSC-M350; Sony) at 0.033-ms intervals, and then captured on a computer. Each observation area was 120 × 90 μm (width × height) per field, and the number of cells in three randomly selected areas was counted every 5 min after starting an experiment. Each experiment was repeated four times.

### Statistical analysis

Statistical analysis (the Student’s *t*-test) was performed to evaluate significant differences in all experiments. Analysis was done by Microsoft Excel (Microsoft, WA, USA) and OriginPro 8 (OriginLab, MA, USA).

## Results

### Effect of MBL on the motility of *Salmonella*

**Fig 1:**
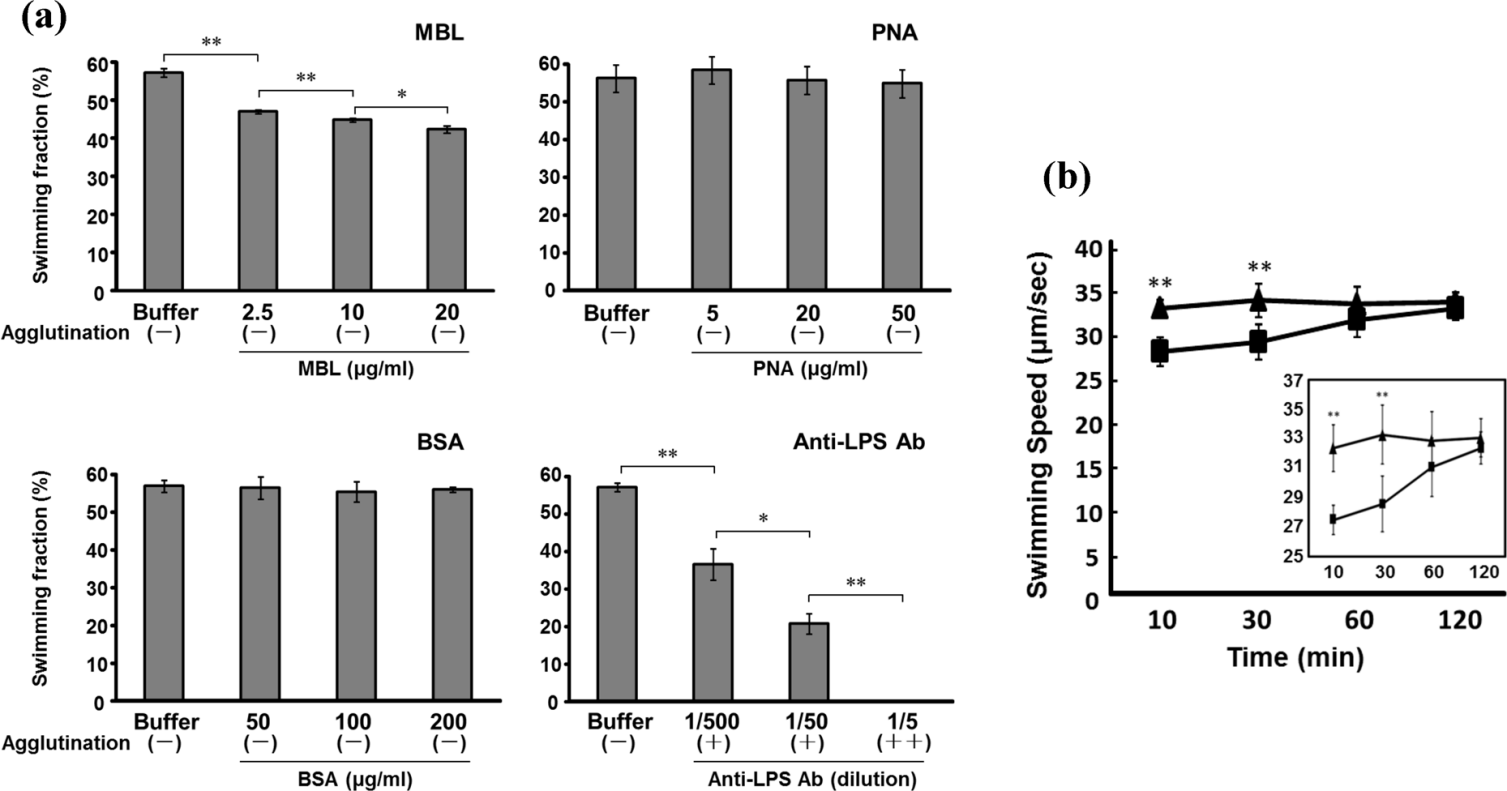
Motility of *Salmonella* was affected by the addition of MBL. (a) Swimming fraction. More than 100 cells were analyzed 30 min after starting an experiment. The average values of three independent experiments are shown. The agglutination was observed under the microscope and evaluated as shown in parenthesis as follows: −, no agglutination; +, partially agglutinated but some cells displaying motility; ++, completely agglutinated (b) Time course of the swimming speed measured in the presence of 2.5 μg/ml MBL. The time displayed on the horizontal axis is one after the addition of MBL. Rectangles and triangles represent MBL-treated cells and the control group, respectively. The average values of more than 100 cells are shown. Vertical lines denote the standard deviation. Statistical analysis (the Student’s *t*-test) was performed to evaluate the significance of the difference from the data of the control at each concentration (*P < 0.05, **P < 0.01).

To test the effect of MBL on the motility of *Salmonella*, we analyzed the fraction of swimming cells in the absence and presence of MBL. In the motility buffer without MBL, approximately 55% of the cells displayed the swimming behavior; the others were likely non-motile owing to problems of flagellation or physiological conditions (Fig 1A and S1 Movie). When the physiological concentration (2.5 μg/ml) of MBL was added, the fraction of swimming cells was significantly decreased to 47% and the inhibitory effect on *Salmonella* motility was enhanced with a dose of MBL, in which no agglutination was observed (Fig 1A and S2 Movie). Adding either BSA or PNA to the cell suspension did not affect the *Salmonella* motility regardless of their doses (Fig 1A). Although anti-O antigen (LPS) antibody reduced the motile fraction, the inhibitory effect involved agglutination of the cells (Fig 1A and S3 Movie). We examined the binding site of MBL in *Salmonella* cells by immunoblotting, enzyme-linked immunosorbent assay (ELISA) and immune-electron microcopy, and confirmed that MBL binds to the cell bodies (particularly LPS structure) but not to the flagella (S1, S2 and S3 Figs). These results suggest that the association of MBL with the cell body disturbed the swimming behavior of *Salmonella* cells.

Fig 1(B) shows the effect of MBL on the swimming speed of *Salmonella*. The swimming speed of the strain st1wt in the motility buffer without MBL was 33.3 μm/s (n = 110) at 30 min after starting an observation. When the cells were treated with 2.5 μg/ml MBL after 30 min, their swimming speeds were significantly reduced to 28.6 μm/s (*P* < 0.05). The swimming speeds of *Salmonella* recovered to the control level within several hours. This indicates that motility inhibition by MBL is temporary in agreement with the previous report showing the inhibitory effect of MBL on spirochete motility [24],.

### Flagellar motor rotation

**Fig 2:**
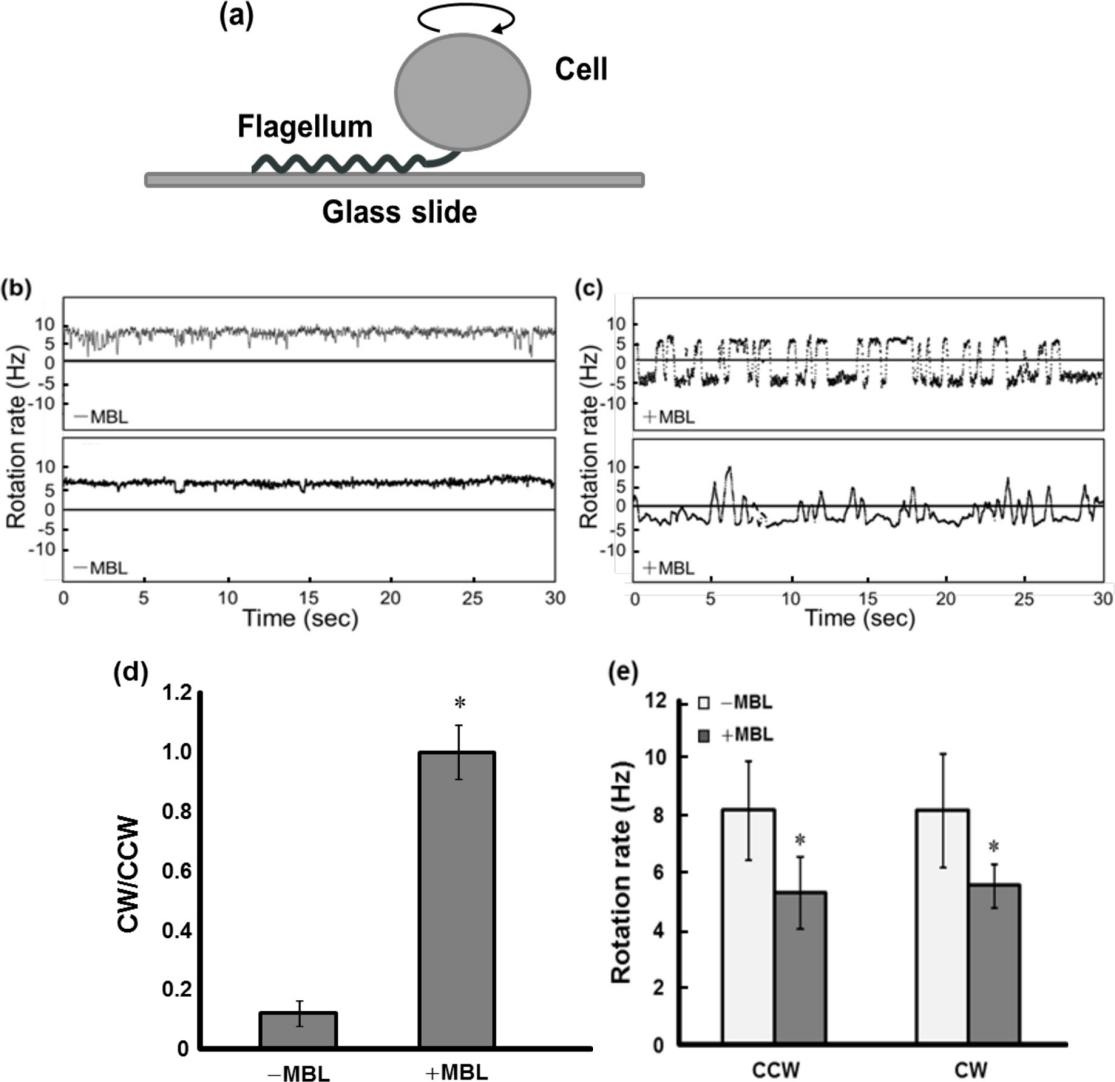
Effect of MBL on single flagellar rotation in wild-type *Salmonella*. (a) Schematic representation of the tethered cell assay. Typical results obtained from independent cells in motility buffer in the absence (b) and presence (c) of MBL are shown. The positive and negative values indicate counterclockwise (CCW) and clockwise (CW) rotations, respectively. (d) Ratio of CW to CCW rotations. Values are the average of 25 flagellar motors. Vertical lines denote the standard deviation. (e) Average rotation rates of *Salmonella* flagella measured 30 min after the addition of MBL to the motility medium. The data are the average values of 21 cells obtained in three independent trials. Vertical lines denote the standard deviation, and asterisks indicate the results of statistical analysis (*P < 0.05).

To evaluate the effect of MBL on *Salmonella* motility more clearly, we analyzed the rotation of a single flagellar motor by the tethered-cell assay (Fig 2A). In the motility medium without MBL, cells tethered on the glass through a single flagellum displayed CCW-biased rotation (Fig 2B and S4 Movie) [13]. The addition of MBL to the motility buffer remarkably increased the reversal frequency of the flagellar motor (Fig 2C and S5 Movie). Although the ratio of CW to CCW rotations (CW/CCW) in the absence of MBL was approximately 0.2 (n = 25), the addition of MBL increased the CW/CCW ratio to about 1.1 (n = 25) (Fig 2D). The rotation rates were also affected by the addition of MBL; the rates of both CCW and CW rotation were reduced to about 65% of those measured in the absence of MBL (Fig 2E). These results indicate that the disturbance of swimming behaviors observed upon addition of MBL was caused by interruption of flagellar rotation.

### Effect of MBL on the intracellular pH

PMF is defined as the sum of the pH difference between the interior and exterior of the cell (ΔpH), and the membrane potential [40, 41]. It is known that a reduction of intracellular pH can induce frequent switching of rotational direction with a decreased rotation rate in bacterial flagella [14, 27, 42]. We thus examined the effect of MBL on intracellular pH using a pH-sensitive fluorescent probe.

**Fig 3:**
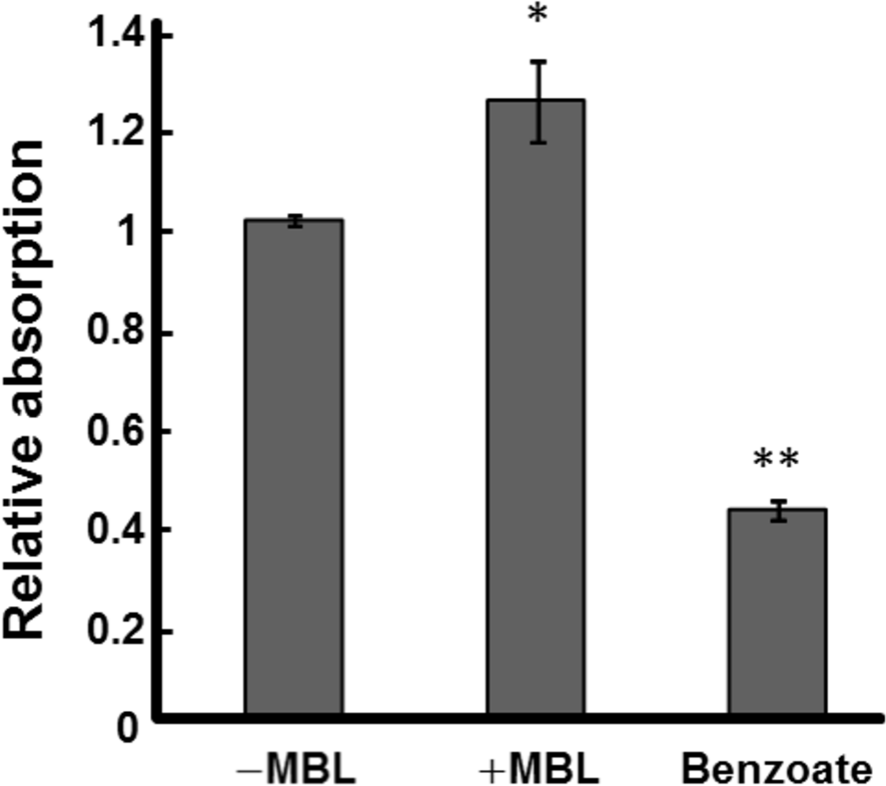
Intracellular pH measured using EGFP. Absorbance of EGFP at 490 nm was determined under each condition. Relative values of absorbance compared to the results obtained in the motility buffer (pH 7.0) without MBL (−MBL) are shown. A decrease in the absorption indicates that the intracellular pH is lowered (see main text). The average values of triplicate experiments are shown. Vertical lines denote the standard deviation, and the Student *t*-test was performed to evaluate the significance of the difference from the result obtained in the absence of MBL (*P < 0.05, **P < 0.01).

The intracellular pH was evaluated by determining the change in the absorption spectra of EGFP: absorption at around 490 nm tends to decrease as pH decreases [34]. We first measured the intracellular pH of *Salmonella* cells in the motility buffer containing benzoate. Weak acids such as acetate and benzoate are known to cross the cytoplasmic membrane in their neutral forms and dissociate protons in the cytoplasm; hence the intracellular pH is decreased by decreasing the extracellular pH in the presence of weak acids [14, 27, 43]. When 20 mM benzoate was added to the motility medium, the fluorescent intensity at 508 nm significantly decreased to about 40% of the values measured in the motility buffer without benzoate, indicating that the intracellular pH of *Salmonella* cells had decreased (Fig 3). In contrast, MBL-treated cells displayed a slight but nonetheless significant increase in fluorescent intensity (*P* < 0.05), indicating that the binding of MBL to *Salmonella* cells increased their intracellular pH.

### Effect of MBL on the membrane potential

**Fig 4:**
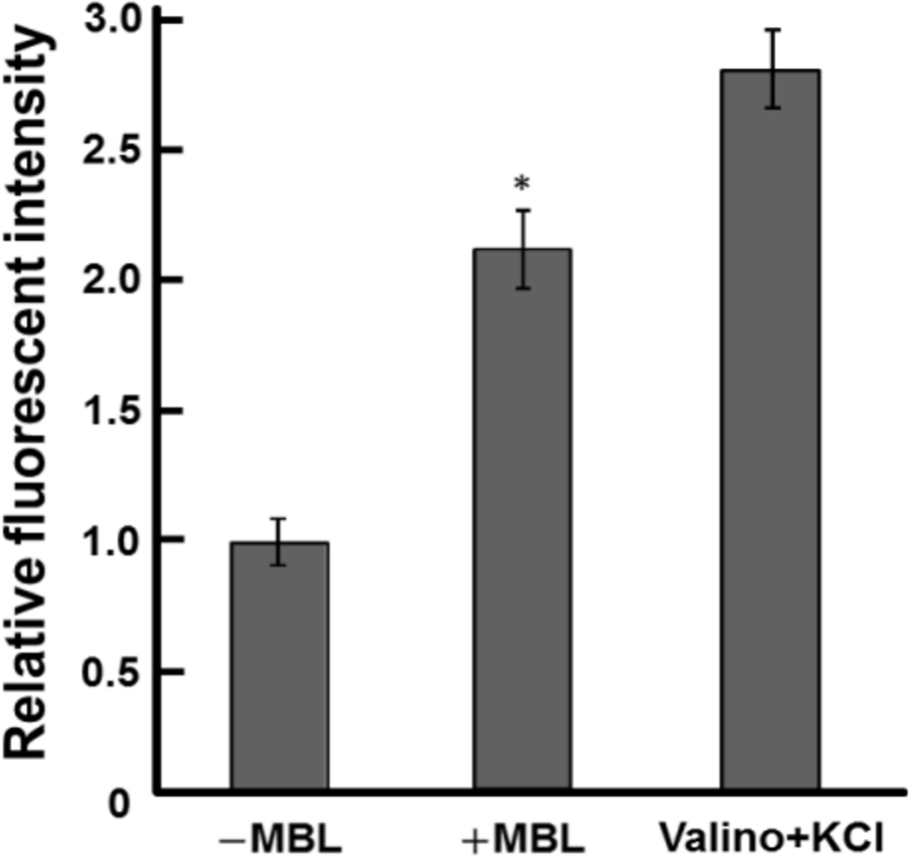
Effect of MBL on the fluorescence intensity of DiBAC_4_(3). An increase in fluorescent intensity corresponds to a decrease in membrane potential. Fluorescent intensities at 517 nm obtained by excitation at 495 nm were determined using a fluorescence plate reader. The values are relative to the fluorescent intensity obtained in the motility buffer without MBL (−MBL). The average values of triplicate experiments are shown. Vertical lines are the standard deviation. The Student *t*-test was performed to indicate a significant difference between the results of −MBL and +MBL (*P < 0.05).

The membrane potential, the other energetic parameter of flagellar rotation, was measured using DiBAC_4_(3) [35]. When DiBAC_4_(3) is added to a cell suspension, the dye distribution in the cytosol increases owing to depolarization of the membrane, resulting in an increase in the fluorescent intensity. Artificial depolarization can be induced by treating cells with a combination of the potassium ionophore valinomycin and KCl [36, 38]. We observed that the fluorescence intensity of DiBAC_4_(3) obtained from cells treated with 20 μM valinomycin and 150 mM KCl increased by about 2.5 times compared to the results obtained from cells in the motility medium without MBL (Fig 4). The MBL treatment also significantly increased the fluorescent intensity, by about 2-fold compared to the results obtained in the absence of MBL (Fig 4). These results indicate that the association of MBL with *Salmonella* cells decreases their membrane potential.

We next tested whether the reduction of membrane potential decreases the rotation rate and increases the reversal frequency of tethered cells by using valinomycin and KCl. Bacteria treated with valinomycin and KCl showed a reduction in their flagellar rotation rates (S4 Fig), and these changes were consistent with the observations in the presence of MBL (Fig 2E). However, the switching frequency was not affected by the reduction of membrane potential (S4 Fig). Therefore, another explanation is required for the highly frequent reversal frequency observed in the presence of MBL.

### Effect of MBL on chemotaxis

**Fig 5:**
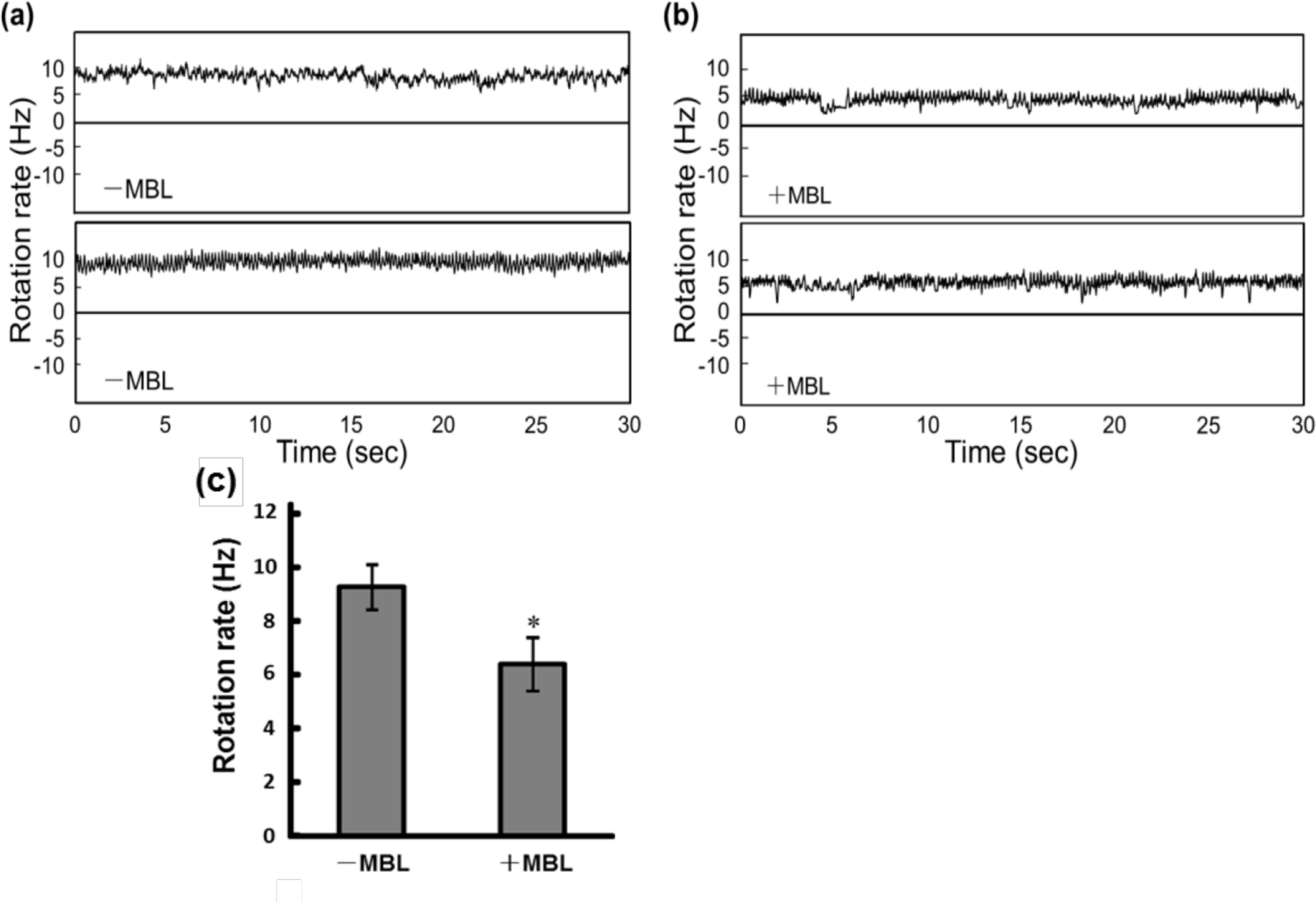
Effect of MBL on flagellar rotation in the chemotaxis-deficient mutant of *Salmonella*. All of the experimental conditions are the same as those described for the assay of the wild-type strain (Fig 2). Typical results obtained from independent cells in motility buffer in the absence (a) and presence (b) of MBL are shown. (c) The average rotation rates. Vertical lines denote the standard deviation, and asterisks indicate the results of statistical analysis. Data represent the average value of 19 cells obtained in three independent trials (*P < 0.05).

The reversal frequency of the flagellar motor is regulated via the chemotactic signaling pathway [44, 45]. To examine whether the binding of MBL to *Salmonella* cells affects their chemotaxis system, we analyzed the effect of MBL on the flagellar rotation of *Salmonella* strain SJW3076, which lacks the genes responsible for signal transduction in chemotaxis, resulting in exclusive CCW flagellar rotation [26]. The tethered cell assays showed that the reversal frequency of SJW3076 flagella was not affected by the addition of MBL (Fig 5A and 5B), whereas their rotation rates were decreased by the addition of MBL (Fig 5C). These results suggest that the increase in the phenomenon of the effect of MBL addition on flagellar reversal frequency occurs via the chemotaxis pathway.

**Fig 6:**
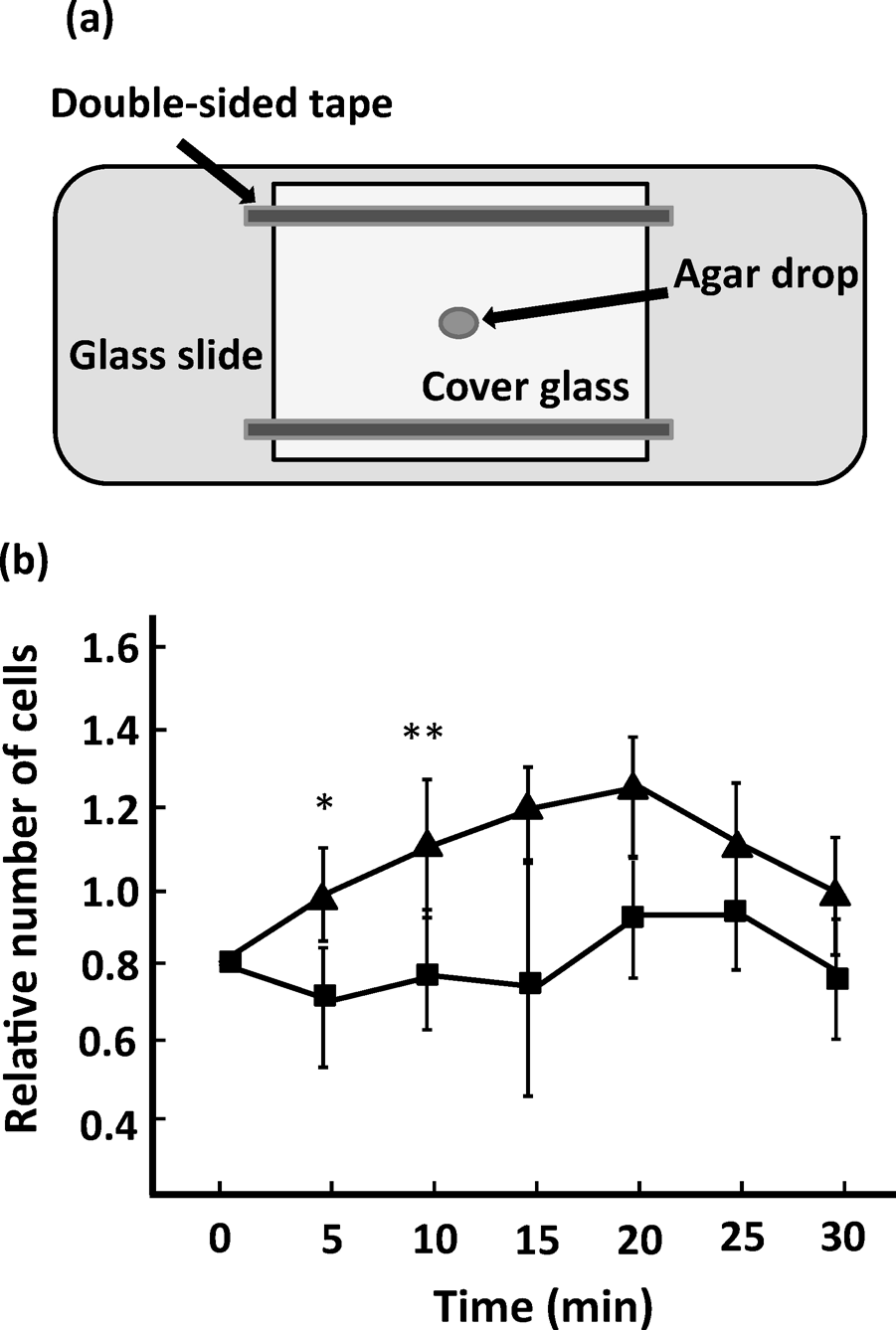
Chemotaxis assay of *Salmonella*. (a) Schematic presentation of the flow chamber used in the microscopic agar drop assay. (b) An attractive response of *Salmonella* to the agar drop containing 10 mM serine. Squares and triangles represent the results obtained in the presence and absence of MBL, respectively. The values represent the ratios of the number of cells near the agar drop at each time point relative to that at 0 min, immediately after starting the assay. Data are the average values of four independent experiments. Vertical lines are the standard deviation and the statistical analysis (the Student’s *t*-test) was preformed to evaluate the significance of the difference from the results of the control (*P < 0.05, **P < 0.01).

To verify whether the reversal frequency increased by MBL influences the chemotactic behavior of *Salmonella* strain st1wt cells, we conducted a microscopic agar drop assay (Fig 6A), which enables evaluation of migration toward attractants or away from repellents in a range of bacteria [39]. In the motility buffer without MBL, the cells rapidly gathered around the agar drop containing an attractant serine (Fig 6B), indicating a positive chemotactic response [18]. In contrast, cells treated with MBL displayed an attractive response to the attractant but distinctly took a much longer time than observed in the absence of MBL treatment, indicating that MBL could interfere with the chemotaxis of *Salmonella*.

## Discussion

Interactions between MBL and microorganisms have been widely investigated from an immunological perspective. A major role of MBL is to recognize carbohydrate patterns on the surface of wide range of microorganisms, resulting in activation of lectin pathway of the complement system. MBL may also cooperate with phagocytic cells to enhance the opsonization of pathogens [8]. It was recently reported that the binding of MBL to spirochetes inhibits their motility [24]. Because bacterial motility is known to strongly correlate with virulence, this finding suggests a novel contribution of MBL to host immunity against infections of microorganisms; namely, MBL is proposed to be responsible for not only triggering innate immunity but also indirectly attacking pathogens by impairing their motility. However, it is unclear precisely how the binding of MBL to bacteria disturbs their movements. In this study, we showed that the inhibitory effect of MBL on *Salmonella* motility is caused by a direct influence on the driving forces required for flagellar rotation, and possibly influencing the signaling pathway of chemotaxis.

When a physiological concentration of MBL was added to the medium, the intracellular pH increased (Fig 3), whereas the membrane potential decreased (Fig 4). These results are similar to a phenomenon previously observed in *Escherichia coli*, in which elevation of the intracellular pH due to the addition of a weak organic base to the medium induced an increase in the membrane potential [43]. The observed increase in intracellular pH by the addition of MBL should increase ∆pH (Fig 3); however, the sum of ∆pH and membrane potential, the driving force for motility PMF, appears to be decreased, given that the decrease in the membrane potential was larger than the elevation in intracellular pH (Fig 4). Although quantitative measurements of energetic parameters are required, these results suggested that MBL interfered with the motility of *Salmonella* by reducing PMF.

The binding of MBL to the cell surface might impede functions of the transmembrane ion pumps and ion channels responsible for maintaining the ion-concentration gradient across the membrane. The surface charge of pathogens could be lost by membrane depolarization, which would facilitate phagocytosis of white blood cells (phagocytic cells), as shown for a part of opsonin and antibodies [46, 47]. The ion-concentration gradient and membrane potential are important factors for the biological activities of bacteria besides their motility, including cell division [48], secretion of toxins [38], and efflux of antibiotics [49]. Our findings suggest the possibility that depolarization of pathogens by MBL could secondarily contribute to the host immune response in parallel with the generally known lectin pathway mechanism.

We found that MBL binding considerably increases the reversal frequency of flagella. The rotational direction of flagella is regulated by a chemotaxis signaling pathway, which is triggered by the binding of substrates to transmembrane receptors known as methyl-accepting chemotaxis proteins (MCPs) [44, 45]. When a cell senses unfavorable substrates such as alcohols via MCPs, a response regulator protein, CheY, is phosphorylated and then the flagellar motor is rotated in CW owing to the association of phosphorylated CheY (CheY-P) with the motor basal body. In contrast, the binding of attractants such as sugars and amino acids to MCPs decreases the level of CheY-P, which in turn increases the probability of CCW rotation. Because MBL did not alter the reversal frequency of flagellar rotation in the mutant *Salmonella* strain lacking the genes responsible for chemotaxis (Fig 5A and 5B), the binding of MBL to the cell surface (S1 Fig) is considered to affect the function of MCPs and then lead to disordered chemotactic behavior. We demonstrated that the migration of *Salmonella* toward the attractant was impaired in the presence of MBL (Fig 6B). The reduction of the swimming speed and the flagellar rotation rate by the addition of MBL (Figs 1B and 2E) may also have contributed to the reduced response rate. Because directed migration is important for pathogenic bacteria to accomplish their infection [50, 51], the effect of MBL on chemotaxis appears to be a feasible mechanism to assist the immunological responses.

The inhibitory effect of MBL on the bacterial motility would be considerably minor part of innate immunity in comparison with immunoglobulins paralyzing bacteria by agglutination (Fig 1A). However, perturbing energetic parameters and directed migrations of pathogens is possibly involved in processes of innate, pre-immune response. Further investigations should help to elucidate precisely how the MBL binding affects the functioning of molecular complexes in the cell membrane, such as ion pumps/channels and MCPs.

## Supporting Information

S1 Fig Confirmation of the binding of MBL to *Salmonella* cells by immunogold labeling and transmission electron microscopy. MBL, anti-MBL rabbit antibody, and colloidal gold particle (10-nm diameter)-conjugated anti-rabbit IgG antibody were used as a primary, secondary, and tertiary antibody, respectively. In a control experiment shown in (a), cells were treated only with the secondary and tertiary antibodies. (b) Cells treated with MBL showed colloidal gold particles on the cell-body surface.
(TIFF)

S2 Fig Confirmation of the MBL binding to *Salmonella* lipopolysaccharide by enzyme-linked immunosorbent assay (ELISA). *Salmonella* LPS was diluted to 5 μg/ml in chloroform-ethanol (1:10, v/v) solution. 100 μl of the solution was added to each well of a 96 wells EIA microplate (Linbro/MP Biochemicals) and evaporated for dryness by incubating overnight at room temperature. Free binding sites were blocked with 200 μl of PBS-Tween with 3% skim milk per well for 60 min at 37°C. We split the experiments into three groups as follows: LPS was treated with MBL for 60 min then reacted with anti-MBL rabbit antibody (left); LPS was tested with rabbit monoclonal antibody specific for the O antigen of O4 (middle); LPS was tested with anti-MBL antibody as a control (right). Additional blocking treatment with H_2_O_2_ in methanol (3%) was conducted to avoid potential nonspecific signal. After being washed seven times with PBS-Tween, all groups were incubated with 100 μl of secondary antibody (1:4,000 dilution of anti-rabbit IgG antibody horseradish peroxidase [HRP]-conjugated) per well at 37°C for 60 min. After properly washing, the OD_450_ was determined with a plate reader (PowerScan HT, DS Pharma Biomedical, Osaka, Japan). All experiments were performed in triplicate, and the averages for triplicates were plotted as bar graph with standard deviation. Student’s *t*-test was performed for evaluating significant difference from the control (*P < 0.05, **P < 0.01).
(TIFF)

S3 Fig Confirmation of the binding of MBL to the *Salmonella* flagella by immunoblotting. Bacterial flagella were detached from cell body by using vortex, the supernatant contains flagellin was recovered from bacteria by applying centrifugation (8,000 ×*g*, 2 min). Then ultra-centrifugation (126,000 ×*g*, 60 min) was performed to isolate flagellin from supernatant, the collected flagellin were confirmed by applying SDS- Polyacrylamide gel electrophoresis and the use of proper molecular marker. The flagellin were separately loaded onto two polyacrylamide gels and further blotted onto two PDVF membranes. Membrane (a) was probed with anti-flagellin rabbit antibody, and the anti-rabbit IgG antibody (horseradish peroxidase [HRP]-conjugated) to detect the expect signal of bacterial flagellin. Membrane (b) was first treated with MBL for 1 hour and later probed with anti-MBL rabbit antibody, anti-rabbit IgG antibody (HRP-conjugated) sequentially. Results of immunoblotting were evaluated with a luminol-base chemiluminescence assay. All experiments were performed in triplicate and showed similar results. These results suggest that MBL does not bind to the flagella of *Salmonella*.
(TIFF)

S4 Fig Effect of a decrease in the membrane potential on rotation of the tethered cells. The cells were treated with valinomycin and KCl. The reduction of the membrane potential decreased the rotation rates of tethered cells but did not affect their reversal frequencies.
(TIFF)

S1 Movie *Salmonella* cells in the motility buffer. (AVI)

S2 Movie *Salmonella* cells treated with 20 μg/ml of MBL. (AVI)

S3 Movie *Salmonella* cells treated with a 1/5 dilution of anti-LPS antibody. (AVI)

S4 Movie A tethered cell in the motility buffer. (AVI)

S5 Movie A tethered cell treated with 2.5 μg/ml of MBL. (AVI)
