# Supplementary figures and images for: Mannose-Binding Lectin Inhibits the Motility of Pathogenic Salmonella by Affecting the Driving Forces of Motility and the Chemotactic Response
Source: PLoS One. 2016 Apr 22;11(4):e0154165. doi: 10.1371/journal.pone.0154165 (PMC4841586; doi:10.1371/journal.pone.0154165)

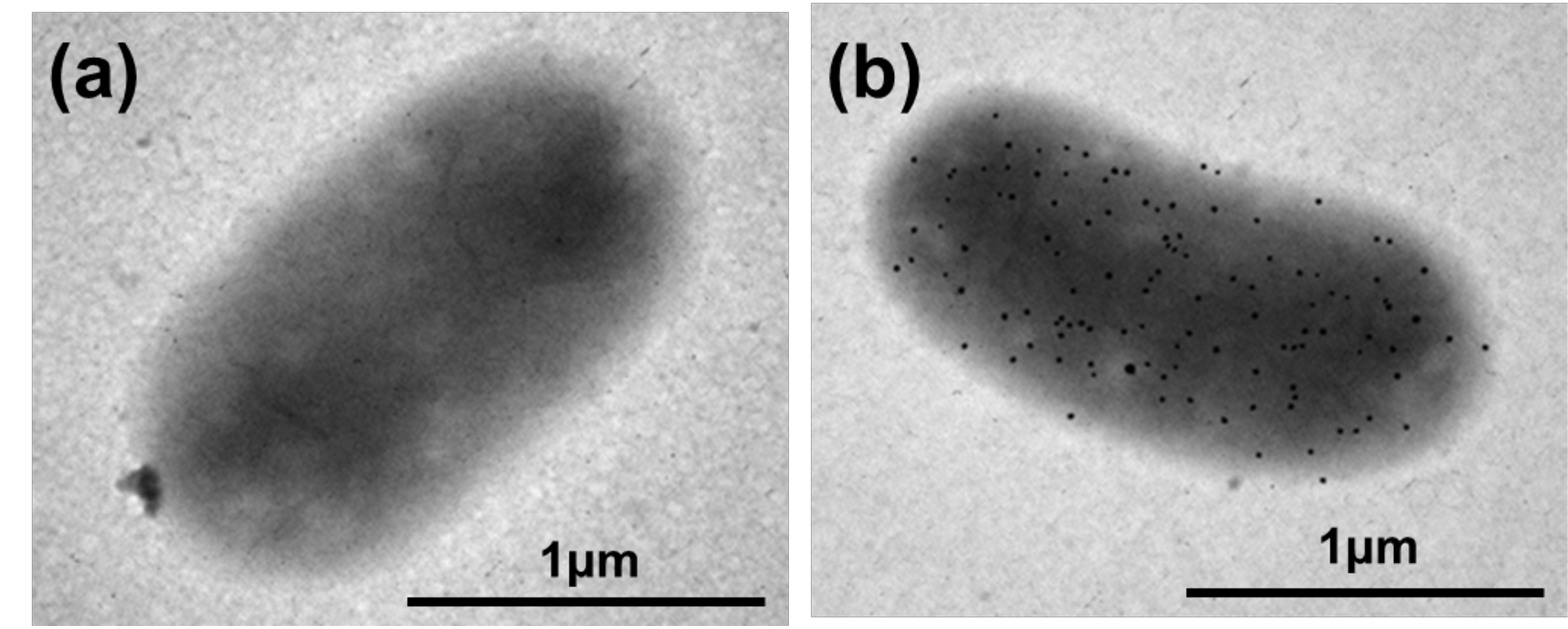

Supplement: S1 Fig — MBL, anti-MBL rabbit antibody, and colloidal gold particle (10-nm diameter)-conjugated anti-rabbit IgG antibody were used as a primary, secondary, and tertiary antibody, respectively. In a control experiment shown in (a), cells were treated only with the secondary and tertiary antibodies. (b) Cells treated with MBL showed colloidal gold particles on the cell-body surface. (TIFF) [file pone.0154165.s001.tiff]

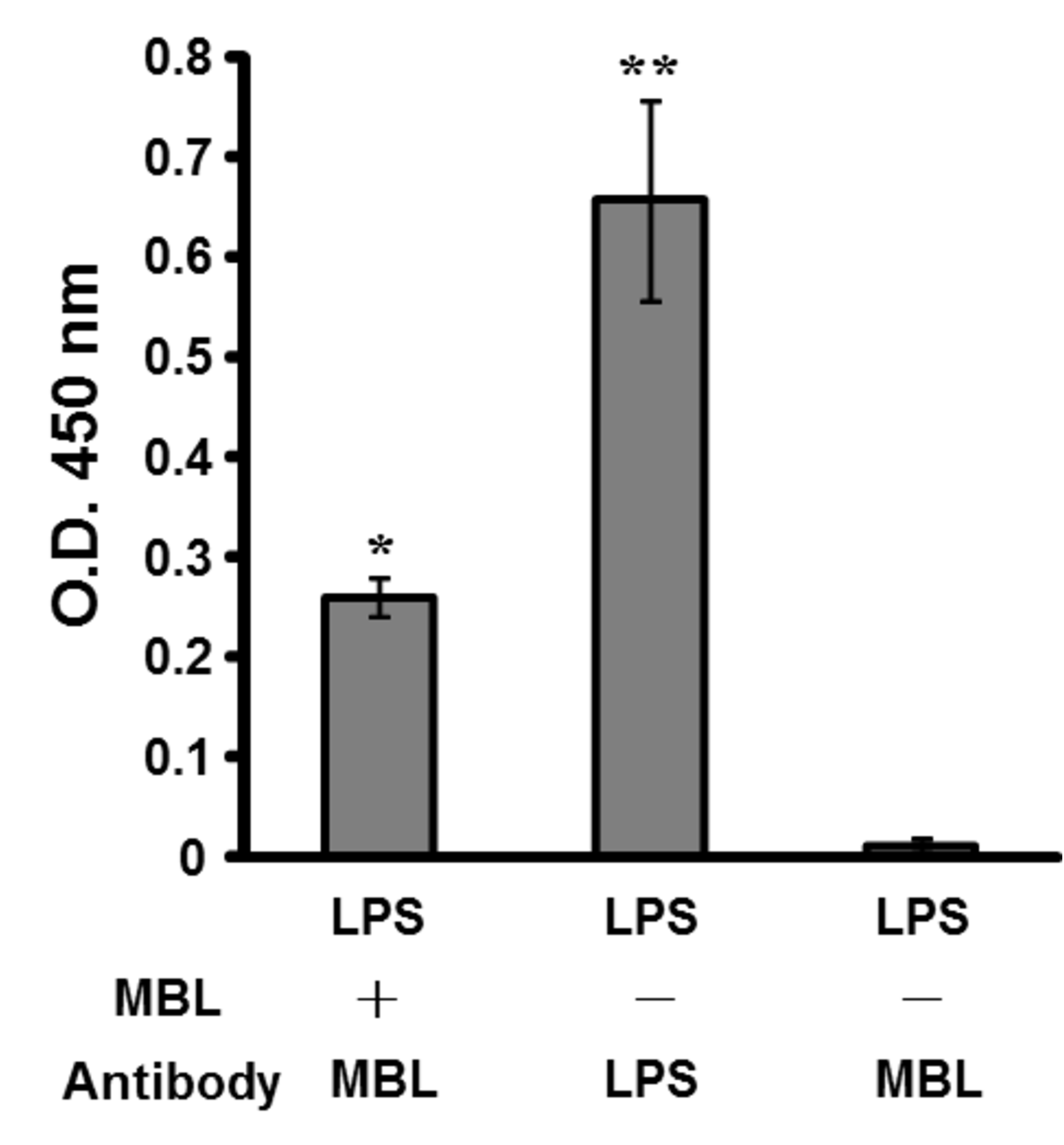

Supplement: S2 Fig — Salmonella LPS was diluted to 5 μg/ml in chloroform-ethanol (1:10, v/v) solution. 100 μl of the solution was added to each well of a 96 wells EIA microplate (Linbro/MP Biochemicals) and evaporated for dryness by incubating overnight at room temperature. Free binding sites were blocked with 200 μl of PBS-Tween with 3% skim milk per well for 60 min at 37°C. We split the experiments into three groups as follows: LPS was treated with MBL for 60 min then reacted with anti-MBL rabbit antibody (left); LPS was tested with rabbit monoclonal antibody specific for the O antigen of O4 (middle); LPS was tested with anti-MBL antibody as a control (right). Additional blocking treatment with H2O2 in methanol (3%) was conducted to avoid potential nonspecific signal. After being washed seven times with PBS-Tween, all groups were incubated with 100 μl of secondary antibody (1:4,000 dilution of anti-rabbit IgG antibody horseradish peroxidase [HRP]-conjugated) per well at 37°C for 60 min. After properly washing, the OD450 was determined with a plate reader (PowerScan HT, DS Pharma Biomedical, Osaka, Japan). All experiments were performed in triplicate, and the averages for triplicates were plotted as bar graph with standard deviation. Student’s t-test was performed for evaluating significant difference from the control (*P < 0.05, **P < 0.01). (TIFF) [file pone.0154165.s002.tiff]

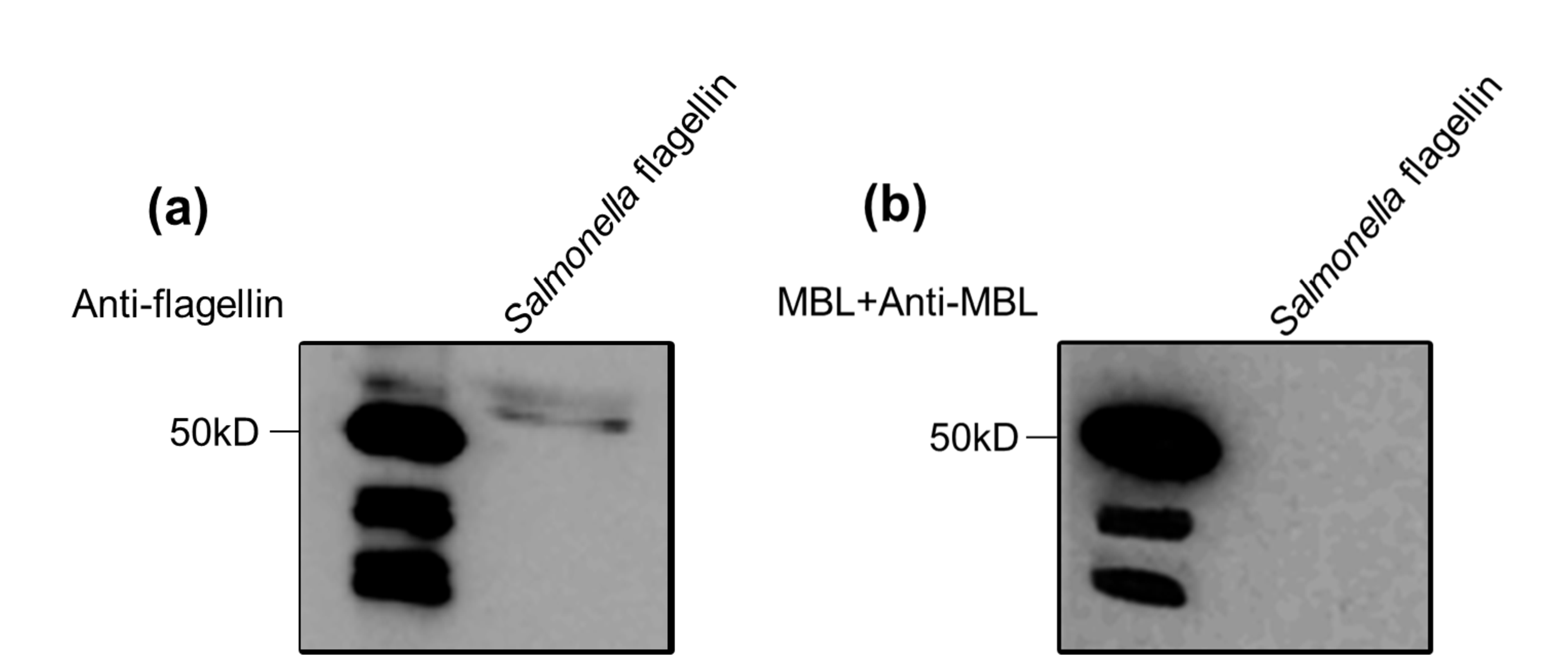

Supplement: S3 Fig — Bacterial flagella were detached from cell body by using vortex, the supernatant contains flagellin was recovered from bacteria by applying centrifugation (8,000 ×g, 2 min). Then ultra-centrifugation (126,000 ×g, 60 min) was performed to isolate flagellin from supernatant, the collected flagellin were confirmed by applying SDS- Polyacrylamide gel electrophoresis and the use of proper molecular marker. The flagellin were separately loaded onto two polyacrylamide gels and further blotted onto two PDVF membranes. Membrane (a) was probed with anti-flagellin rabbit antibody, and the anti-rabbit IgG antibody (horseradish peroxidase [HRP]-conjugated) to detect the expect signal of bacterial flagellin. Membrane (b) was first treated with MBL for 1 hour and later probed with anti-MBL rabbit antibody, anti-rabbit IgG antibody (HRP-conjugated) sequentially. Results of immunoblotting were evaluated with a luminol-base chemiluminescence assay. All experiments were performed in triplicate and showed similar results. These results suggest that MBL does not bind to the flagella of Salmonella. (TIFF) [file pone.0154165.s003.tiff]

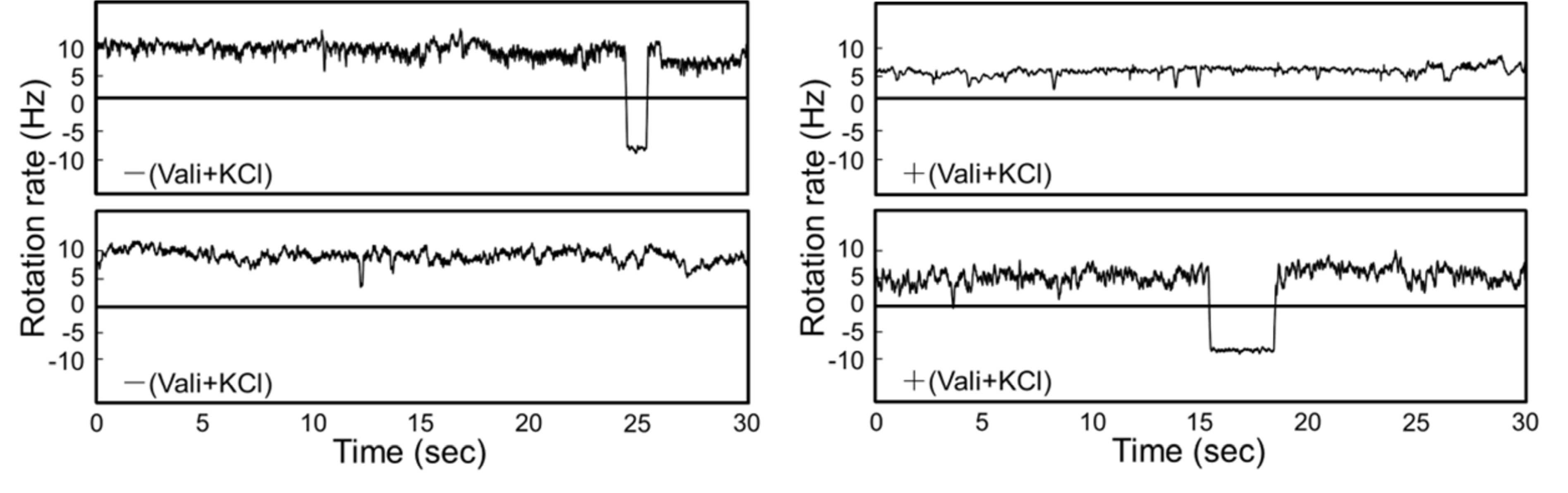

Supplement: S4 Fig — The cells were treated with valinomycin and KCl. The reduction of the membrane potential decreased the rotation rates of tethered cells but did not affect their reversal frequencies. (TIFF) [file pone.0154165.s004.tiff]
